# Free Space Optics Transmission Performance Enhancement for Sustaining 5G High Capacity Data Services

**DOI:** 10.3390/mi13081248

**Published:** 2022-08-03

**Authors:** Mustafa Kamal, Jahanzeb Khan, Yousaf Khan, Farman Ali, Ammar Armghan, Fazal Muhammad, Nasim Ullah, Sattam Alotaibi

**Affiliations:** 1 Department of Electrical Engineering, Iqra National University, Peshawar 25124, Pakistan; mustafa.kamal1@gmail.com (M.K.); jehanzeb.khan@inu.edu.pk (J.K.); 2 Department of Electrical Engineering, University of Engineering and Technology, Pesawar 25000, Pakistan; y.khan@uetpeshawar.edu.pk; 3 Department of Electrical Engineering, Qurtuba University of Science and IT, Dera Ismail Khan 29050, Pakistan; drfarmanali.optics@qurtuba.edu.pk; 4 Department of Electrical Engineering, College of Engineering, Jouf University, Sakaka 72388, Saudi Arabia; aarmghan@ju.edu.sa; 5 Department of Electrical Engineering, University of Engineering Technology, Mardan 23200, Pakistan; fazal.muhammad@uetmardan.edu.pk; 6 Department of Electrical Engineering, College of Engineering, Taif University, Taif 21944, Saudi Arabia

**Keywords:** nonlinear interference, co-channel interference, free space optics, free space pointing errors

## Abstract

Enhanced bandwidth issues for 5G system are fruitfully resolved by organizing free space optics (FSO) communication frameworks. The high bandwidth, the maximum number of channel transmission requirements, and high data rate have been boosted during the last years because of the COVID-19 pandemic. The online services and digital applications have increased pressure on installed optical network models. In addition, the optical networks with high capacity transmission produce nonlinear distortions, which degrade system efficiency. This paper presents a mixed FSO and fiber network to tackle the factors of nonlinearities and enrich the system capacity and range. Furthermore, the issues related to radio frequency, FSO pointing, and co-channel interference are considered in this work. The theoretical and simulation structures are validated using advanced measuring parameters, such as bit error rate (BER), peak to average power ratio (PAPR), cumulative distribution function (CDF), and outage probability. The nonlinear factors are addressed successfully, and the capacity is developed from current models. Finally, the proposed model’s limitations and future direction are discussed in this paper.

## 1. Introduction

Recent developments in online marketing have enhanced the value of free space optics (FSO), purposing to improve the capacity and transmission accuracy of communication systems [1]. Low cost installation, improved security, and colossal bandwidth are the critical properties of FSO; therefore, communication system FSO based design is widely used in terrestrial and deep space applications [2]. The main structure of FSO is linked with the setup of an optical network; it takes inputs from laser based transmitters and is received at the photodiode based receiver [3]. Hence, the achievements of FSO can be further enhanced with the joint framework of optical networks. With the help of this structure, the issues in existing FSO models, such as radio frequency (RF) related matters, FSO pointing, and co-channel interference impairments, can be organized efficiently. In addition, the performance of 5G services can be improved by applying the composite structure of FSO and optical networks [4]. In this paper, the outage probability and commutative, distributive function (CDF) calculations are optimized for FSO and optical network based communication links. The simulation and analytical approach are estimated in terms of peak to average power ratio (PAPR) and bit error rate (BER).

### Previous Work and Background

Several research groups around the globe have presented different algorithms and techniques to improve FSO performance. In [5], the authors have shown recent progress on FSO technology and the factors that interpret the outcomes. The study in [6] evaluates the performance of the FSO system in diverse geographical locations. The channel induced limitations are mitigated using orthogonal frequency division multiplexing (OFDM) and digital signal processing (DSP) techniques. A. Bekkali et al. [7] suggested a full duplex and all FSO transceivers and evaluated its performance. The quality factor and electrical power were investigated for FSO links in [8], and simulation analyses were performed using optisystem. Intensity modulation and direct modulation (IM/DD) FSO link were studied in [9] in terms of bit error rate (BER). Ref. [10] explained the OFDM mode division multiplexing (OFDM-MDM) based FSO transceiver. The dust effect is estimated using signal to noise ratio (SNR) and total power as key measuring parameters. The impact of sandstorm conditions was analyzed in [11] for FSO links. The backhaul network was introduced in [12] for a 5G based FSO system, and the performance was evaluated and compared with a conventional FSO link. In [13], the role of the FSO framework was investigated for the next generation satellite communication system. However, the COVID-19 pandemic has given a push to online application and marketing services, which has overburdened the already installed FSO setups. In this paper, the combined structure of the optical network and FSO is introduced to enrich the capacity and transmission accuracy. This mechanism has fruitfully minimized the impacts of FSO pointing and co-channel interference disabilities. This paper is organized as follows. Section 2 consists of an analytical investigation, the proposed mixed FSO and optical network is elaborated in Section 3. Section 4 discusses the results and discussion of the simulation analysis, and, finally, the mixed FSO and optical network model is summarized in Section 5.

## 2. Analytical Modeling

The mixed FSO and fiber link system is introduced in this paper, purposing to minimize the nonlinear issues and FSO related impairments such as RF alignment issues, FSO pointing errors, and co-channel interference. This section includes the analytical calculations for the proposed FSO and optical systems. The channel model of FSO is defined [14,15] as

(1) $$\begin{array}{c} F_{ch}=\psi_{a}\psi_{p}\psi_{l}. \end{array}$$

where $F_{ch}$ presents the FSO channel, $\psi_{a}$ is the atmospheric turbulence loss, $\psi_{l}$ is the geometric loss, and $\psi_{p}$ is the FSO pointing issues. Three components are considered for optical signal transmission [16]: (1) line of sight component, (2) line of sight coupled with the scattered component, and (3) independent scattered component. The power distribution function (pdf) for free space is expressed [17,18,19,20] as

(2) $$\begin{array}{c} f_{F_{ch}}\left(F_{ch}\right)=B\Sigma_{n=1}^{\beta_{m}}a_{n}F_{ch}^{\frac{\alpha_{m}+n}{2}-1}N_{\alpha -n}\left(2\sqrt{\frac{\alpha_{m}\beta_{m}F_{ch}}{\gamma \beta_{m}+P^{\prime }}}\right) \end{array}$$

and *B* means

(3) $$\begin{array}{c} B=\frac{2\alpha_{m}^{\alpha_{m}/2}}{\gamma^{1+\alpha_{m}/2}\varphi \left(\alpha_{m}\right)}{\left(\frac{\gamma \beta_{m}}{\beta_{m}+P^{\prime }}\right)}^{\beta_{m}+\alpha_{m}/2} \end{array}$$

where $\alpha_{m}$ is the large scale scattering process, $\beta_{m}$ is the fading parameter, γ is the independent scattering component, and φ(.) represents gamma function. The $p^{\prime }$ is defined as

(4) $$\begin{array}{c} p^{\prime }=p+2\tau b_{0}, \end{array}$$

where the *p* is the power for the first line of sight component, and $2\tau b_{0}$ is the coupled line of sight and scattered component. The parameter $a_{n}$ is further explained [21] as

(5) $$\begin{array}{c} a_{n}=\left[\begin{array}{c} \beta_{m}-1\\ n-1 \end{array}\right]\frac{{\left(\gamma \beta_{m}+p^{\prime }\right)}^{1-\frac{n}{2}}}{(n-1)!}{\left(\frac{p^{\prime }}{U}\right)}^{n-1}{\left(\frac{\alpha_{m}}{\beta_{m}}\right)}^{\frac{n}{2}} \end{array}$$

where $\beta_{m}$ is the fading element, and $\alpha_{m}$ is the effective number of large scale scattering process. The FSO system performance is conditioned by the transceiver and structural ways; this leads to FSO pointing impairments, and it is calculated in terms of PFD [22,23] as

(6) $$\begin{array}{c} f_{\psi_{p}}=\frac{u^{2}}{A_{0}^{u^{2}}}{\left(\psi_{p}\right)}^{u^{2}-1},0\leq \psi_{p}\leq A_{0} \end{array}$$

where $A_{0}$ is integrated optical power function, and *u* is related to jitter deviation and equal to $\frac{\omega_{z}}{2\sigma^{2}}$. The width of the data carrying laser beam is denoted by $\omega_{z}$. The statistical analysis of FSO pointing errors, turbulence fading, and co-channel interference is expressed [24,25] as

(7) $$\begin{array}{c} f_{F_{ch}}\left(F_{ch}\right)=\int f_{F_{ch}/\psi_{a}}\left(F_{ch}/\psi_{a}\right).f_{\psi a}\left(\psi_{a}\right)d\psi_{a} \end{array}$$

In Equation (7), the $f_{F_{ch}/\psi_{a}}\left(F_{ch}/\psi_{a}\right)$ declares the conditional probability. By substituting Equation (1) to Equation (6) into Equation (7), the CDF of the N channel is defined as

(8) $$\begin{array}{c} f_{F_{ch}}\left(F_{ch}\right)=\frac{u^{2}B}{2}\Sigma_{n=1}^{\beta_{m}}\left(a_{n}{\left[\frac{1}{A_{m}}\right]}^{\frac{\alpha +n}{2}}\right)G_{2,4}^{3,1}\left(\frac{F_{ch}}{AB_{0}I_{l}}\right) \end{array}$$

where $G_{2,4}^{3,1}$ is the Meijer’s G function. On the transmitter side, multi-pulse position modulation (MPPM) based intensity modulation direct detection system is used for the FSO system. The electrical filter is installed on the receiver side to remove unwanted signals from the original signals. The output of the filtered signal is calculated as

(9) $$\begin{array}{c} y\left(t\right)=R\frac{N}{n}P_{R}\Sigma_{n=0}^{N-1}C_{n}+k\left(t\right) \end{array}$$

The average received optical power, *R*, is the photodetector responsitivity, and k(t) is the additive white Gaussian (AWG); $C_{n}$ is the signal time slot. The transmitter and receiver telescope gains are expressed [18,26,27] as

(10) $$\begin{array}{c} G_{t}=G_{r}={\left(\pi d/\lambda_{k}\right)}^{2} \end{array}$$

where $G_{t}$ is the transmitter gain, $G_{r}$ is the receiver gain, and *d* is the diameter. The receiver signal to noise ratio (SNR) of the FSO system is estimated as

(11) $$\begin{array}{c} SNR\left(F_{ch}\right)=R^{2}P_{t}^{2}{\left(\frac{\eta B_{r}}{\lambda_{k}L}\right)}^{4}\frac{Modlog_{2}Mod}{2M\sigma_{n}^{2}}F_{ch} \end{array}$$

where σ is the variance of channel noise, η is the efficiency, Mod is the modulation order, and M is the number of transceivers. The conditional probability error of the presented integrated optical network and FSO system is calculated as

(12) $$\begin{array}{c} BER\left(F_{ch}\right)=\frac{Mod}{4}erfc\left[Rp_{R}\left(F_{ch}\right)\sqrt{Modlog_{2}Mod}/2\sigma_{k}\right] \end{array}$$

where erfc is the error function. The outage probability of the fading channel is calculated as

(13) $$\begin{array}{c} p_{out}=p\left(SNR\right)\leq SNR \end{array}$$

## 3. Mixed FSO and Optical Network Presented Model

**Figure 1:**
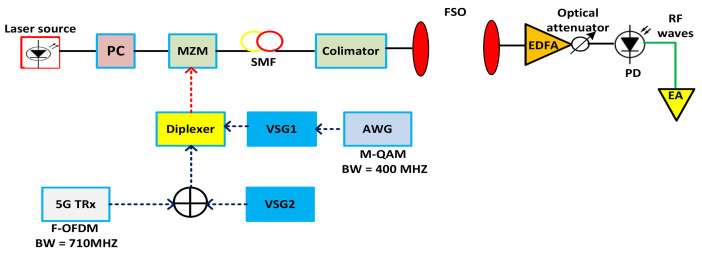
Mixed FSO and optical network based framework for addressing the FSO pointing and co-channel interference.

**Figure 2:**
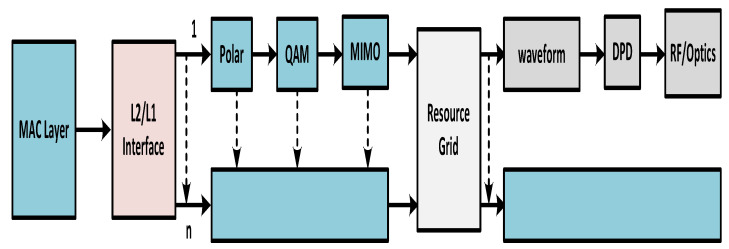
The 5G proposed transceiver for smooth transmission against nonlinear impairments.

The block description of the presented mixed FSO and optical network is depicted in Figure 1. The technologies are converged into a single model, which uses FSO and optical network links to integrate the fronthaul. The 3GPP released 15, 5G frequency standard is used for the presented FSO model, aiming to transmit RF signals. The proposed 5G transceiver generates an F-OFDM signal with 790 MHz. The combiner (3dB insertion losses) is applied to integrate the 790 MHz and 3.5 GHz signals. The received electrical signals are then joined by using a diplexer with M-QAM. The power range is set for two conditions. For normal conditions, the power level for the RF signal is set to 0dBm, whereas the 5 dBm power range is used for M-QAM at 25 GHz because of the RF cable, photodetector (PD), and Mach–Zehnder modulator (MZM). The MZM takes the integrated signals and modulates the laser 10 dBm carrier at 1550 nm. Whereafter these signals are transformed over 15 km single mode fiber (SMF), and, at the collimator, the data are gained for injection to the FSO system. To decrease optical losses and provide a safe environment for FSO, the erbium-doped fiber amplifier (EDFA) is applied. The five-fold magnified optical laser is installed at the receiver, purposing to collimate the optical beam and coupled with an optical cord. The outputs of EDFA are associated with optical attenuation for monitoring the power level. The installed PD converts the optical received signal into electrical form, where the signals are further amplified by an electrical amplifier (EA) with a 20 dB gain. The presented 5G transceiver is displayed in Figure 2, which explains that the data from the MAC layer are sent to the physical layer with the help of the L1 and L2 interface. All the received control information is included for modulation coding and transmitting. The polar code has high correction error capabilities with low complexity. Therefore, it is used as forwarding error correction (FEC) here. The transceiver is designed based on F-OFDM waveform transmission; however, it can also transmit other waveforms such as OFDM. The fundamental goal of the transmitter is to manage the fiber nonlinearities and enhance system accuracy.

## 4. Results and Discussion

**Figure 3:**
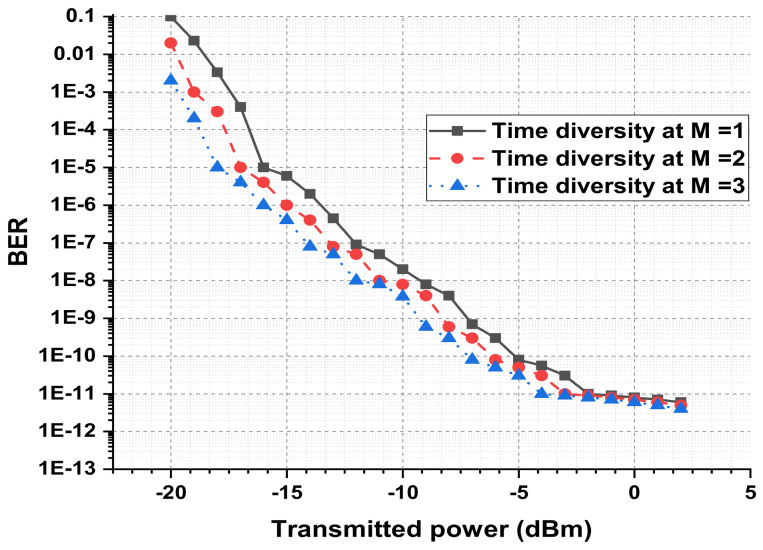
Time diversity analysis in terms of transmitted power and BER.

**Figure 4:**
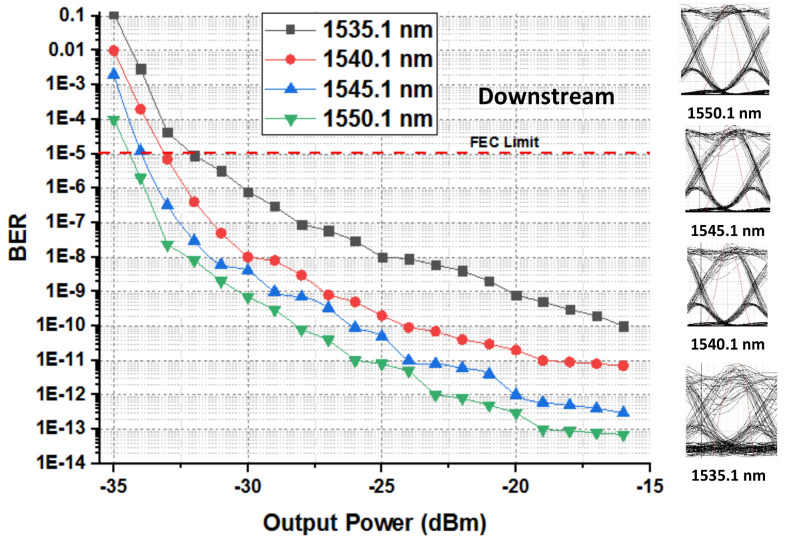
Comparison of different wavelengths using received power and BER.

**Figure 5:**
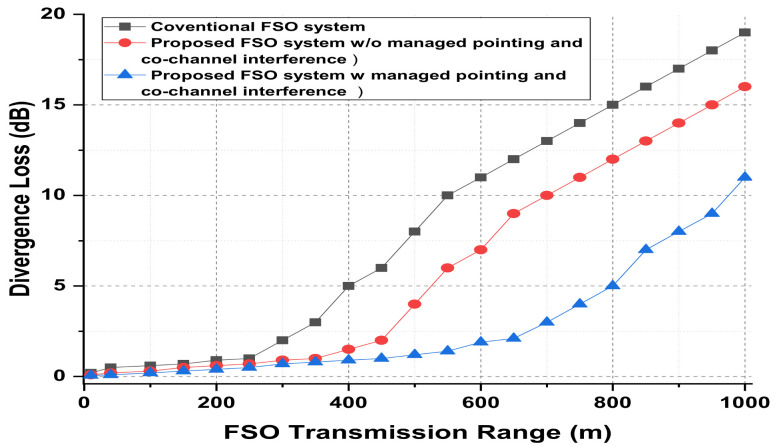
FSO transmission range against divergence loss for analyzing conventional and proposed FSO systems.

**Figure 6:**
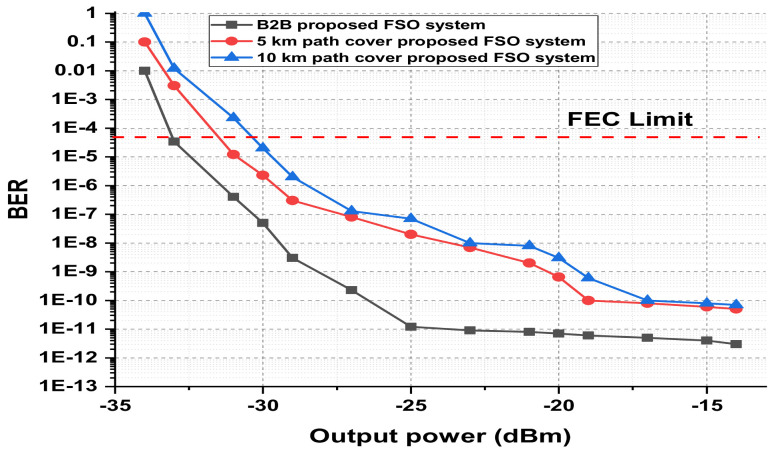
B2B, 5km, and 10 km FSO transmission comparison using output power as a function of BER.

**Figure 7:**
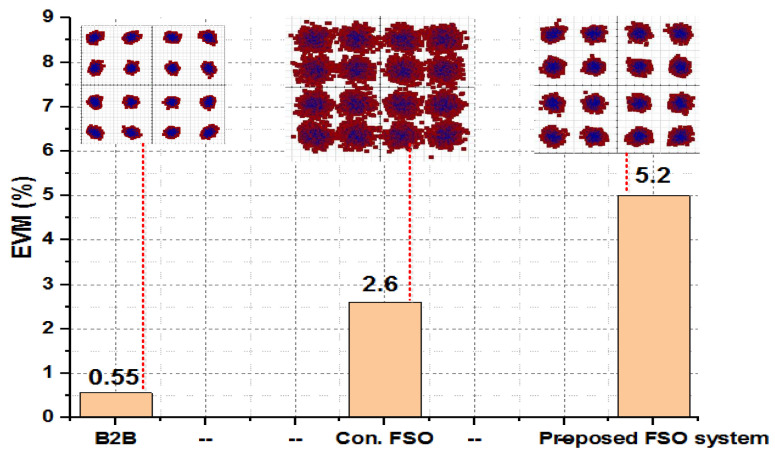
EVM analysis for B2B, conventional, and proposed FSO systems.

**Figure 8:**
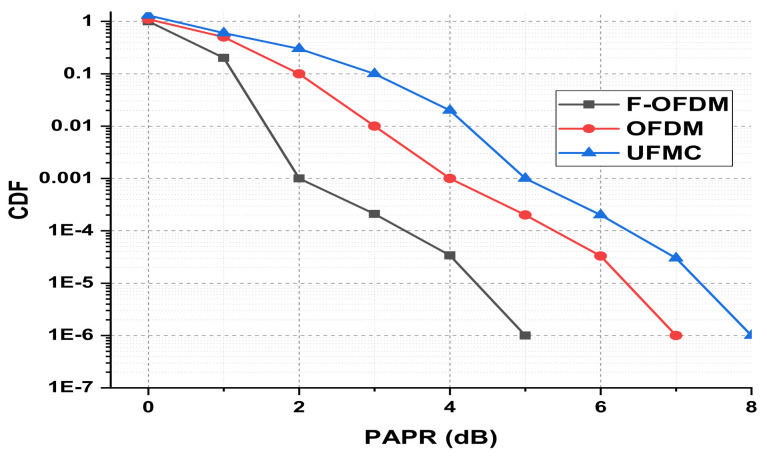
The comparison of F-OFDM based 5G transceiver with OFDM and UFMC in terms of PAPR and CDF.

**Figure 9:**
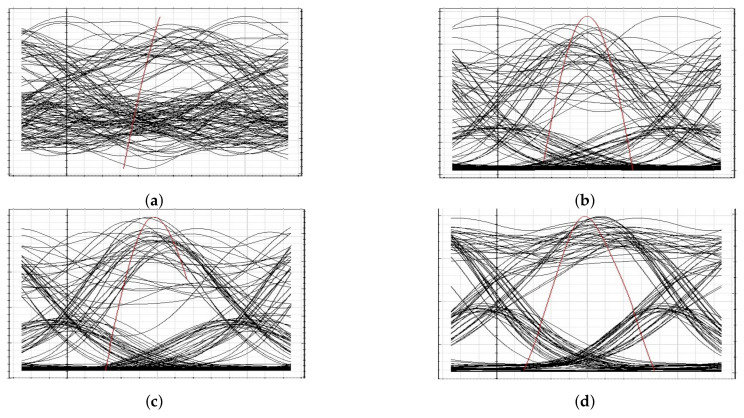
Eye diagram representation for different optical beams: (**a**) single beam, (**b**) double optical beams, (**c**) triple optical beams, and (**d**) four optical beams.

The presented FSO and optical network integrated model is an investigation using optisystem and MATLAB simulation software. The performance is evaluated based on various parameters, such as input power, output power, different wavelengths, FSO transmission range, and divergence losses. The values of the used parameters are listed in Table 1, which are as per practical used models. Figure 3 shows the simulation analysis for different time diversity order (M = 1, 2, 3) using input power and BER. The plotted analysis explains that the BER improves at third order time diversity. The power level from negative to positive decreases the BER value for all the time diversity orders. The diversity with M = 3 gives BER = $10^{-11}$ at a 3 dBm power level, which is considered acceptable efficiency against nonlinearities, FSO pointing errors, and co-channel interference. Thus, the results noted in Figure 3 encourage the system outcomes at diversity order 3. The presented FSO system is tested using different laser wavelengths (1535.1, 1540.1, 1545.1, and 1550.1 nm) based on output power versus BER. The laser signals with 1550.1 nm cross the FEC limit sooner than other transmissions. Maximum BER gap can be noted among 1535.1, 1540.1, 1545.1, and 1550.1 nm signal transmissions. For example, at −18 dBm received power, the BER attained by 1550.1 nm signal is about 1.3 $\times 10^{-13}$; on the other side at 1535.1 nm signal transmission, the BER is recorded above $10^{-9}$. Figure 4 also includes the eye diagram investigation for the tested wavelengths. Figure 5 notes the analysis of the results for FSO transmission range (m) and divergence losses (dB). The estimations compare the conventional FSO system, the proposed FSO system without managed pointing and co-channel interference issues, and with managed pointing and co-channel interference issues. In addition, Figure 5 explains two scenarios: (1) the divergence losses increase with increasing the transmission range; (2) the divergence losses are set higher in the case of the conventional system and presented in the FSO and optical network hybrid system. It can be seen that the presented model fruitfully addresses the FSO pointing and co-channel interference errors. Figure 6 describes the correlation between back-to-back (B2B), 5 km path cover, and 10 km path cover FSO system, applying output power as a function of BER. The performance of B2B is ideal; however, the BER degrades with an increase in length. The outcomes of the FSO system are evaluated using EVM measuring element. This analysis is shown in Figure 7, which clarifies that the presented FSO and optical network mixed model has significant outcomes compared to the conventional FSO system. Figure 8 provides the correlation between the presented F-OFDM 5G transceiver and OFDM and UFMC in terms of PAPR and CDF. The fruitful PAPR is achieved by F-OFDM based signal transmission. The efficiency of the presented model is estimated for various optical beams, as shown in Figure 9. Figure 9a explains the outcomes for single optical beam transmission, Figure 9b shows two beam transmission, and Figure 9c presents the transmission for three optical beams. Figure 9d shows four optical beam transmission outcomes. The comparison of eye diagram estimation in Figure 9 defines that the presented system gives accurate results with multi beam transmissions.

## 5. Conclusions

The combined structure of FSO and optical network using a 5G based F-OFDM transceiver is presented in this paper. It is discussed that fiber nonlinearities, FSO pointing errors, and co-channel interference have limited the optical network and FSO performance. The given model is evaluated theoretically in terms of CDF, outage probability, PAPR, and BER. The simulation results were investigated using input power, output power, FSO transmission range, divergence losses, and CDF. The significant performance of the proposed FSO model was recorded in comparison with current approaches. The encouraged BER is received against fiber nonlinearities, FSO pointing errors, and co-channel interference. In the future, we can further develop the achievements of the presented model by applying machine learning procedures.

## Figures and Tables

**Table 1:** Parameters used for estimating the simulation results.

Parameter	Value
Transmitted power	−20 to 2 dBm
Output power	−40 to −14 dBm
Downstream wavelengths	1535.1 to 1550.1 nm
FSO length	1000 m
Insertion loss	3 dB
F-OFDM signal	790 MHz
EA gain	20 dB

## Data Availability

Not applicable.
